# Open-source computational simulation of moth-inspired navigation algorithm: A benchmark framework

**DOI:** 10.1016/j.mex.2021.101529

**Published:** 2021-09-27

**Authors:** Yiftach Golov, Noam Benelli, Roi Gurka, Ally Harari, Gregory Zilman, Alex Liberzon

**Affiliations:** a Porter School of Environment and Earth Sciences, Tel Aviv University, Israel; b Department of Entomology, The Volcani Center, Bet Dagan, Israel; c School of Mechanical Engineering, Tel Aviv University, Tel-Aviv, Israel; d Department Physics and Engineering Science, Coastal Carolina University, Conway, SC, USA

**Keywords:** Bio-inspiration, Moths, Odor localization, Navigation strategies, Simulation framework

## Abstract

Olfactory navigation is defined as a task of a self-propelled navigator with some sensors capabilities to detect odor (or scalar concentration) convected and diffused in a windy environment. Known for their expertise in locating an odor source, male moths feature a bio-inspirational model of olfactory navigation using chemosensory. Many studies have developed moths-inspired algorithms based on proposed strategies of odor-sourcing. However, comparing among various bio-inspired strategies is challenging, due to the lack of a componential framework that allows statistical comparison of their performances, in a controlled environment. This work aims at closing this gap, using an open source, freely accessible simulation framework. To demonstrate the applicability of our simulated framework as a benchmarking tool, we implemented two different moth-inspired navigation strategies; for each strategy, specific modifications in the navigation module were carried out, resulting in four different navigation models. We tested the performance of moth-like navigators of these models through various wind and odor spread parameters in a virtual turbulent environment. The performance of the navigators was comprehensively analyzed using bio-statistical tests. This benchmark-ready simulation framework could be useful for the biology-oriented, as well as engineering-oriented studies, assisting in deducing the evolutionary efficient strategies and improving self-propelled autonomous systems in complex environments.

• The open-source framework `Mothpy' provides a computational platform that simulates the behavior of moth-like navigators, using two main inputs to be modified by the user: (1) flow condition; and (2) navigation strategy.
• `Mothpy' can be used as a benchmarking platform to compare the performance of multiple moth-like navigators, under various physical environments, and different searching strategies.
• Method name: Mothpy 0.0.1' - an open-source moth-inspired navigator simulator.

## Graphical abstract

**Figure:**
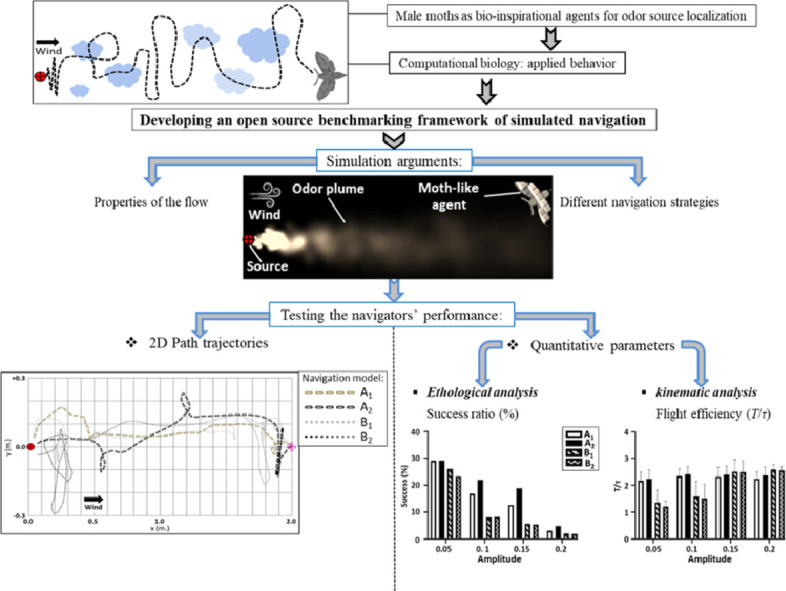

Specifications table

**Table:** 

Subject area:	Engineering
	Agricultural and Biological Sciences
More specific subject area:	Memetic Computing, Applied behavior, Bio-inspired Algorithm
Method name:	`Mothpy 0.0.1' - an open-source moth-inspired navigator simulator
Name and reference of original method:	None
Resource availability:	The open source autopilot as an iceberg tracker can be found here: https://zenodo.org/record/2672828#.YBKAeehvY2w https://doi.org/10.5281/zenodo.2672828

## Introduction

The specialization of organisms in accomplishing different types of tasks has been capitalized as a source of inspiration (i.e. bio-inspiration) in the field of biomimicry. Such is the effectual capability of insects to detect an odor source in airborne milieu for different purposes, such as foraging for food (e.g. nocturnal pollinators), host detection (e.g. in parasitoids) and mate-locating, facilitated by sex pheromones (e.g. bees, flies, beetles, moths) [1], [2]–3]. A pivotal feature in the different types of wind-borne olfactory searching is the ability of a self-propelled agent to successfully locate an odor source with limited sensor capabilities. Numerous odor-locating strategies were proposed and tested in a simulated environment [4,5]. Specific taxa of insects that received major attention in the field of neuroethology are moths [6,7]. Known for their expertise in chemoreception, male moths are highly efficient in locating their conspecific females over long distances by using volatile components, known as sex pheromones, using only local cues (i.e.: without prior information or memory assumptions), in a turbulent environment [8,9]. This is accomplished by using chemo-receptors on their antennae [10], [11]–12] for chemical sensing [13,14], presumably combining visual input, commonly referred to as `optomotor anemotaxis' [15]. The navigational behavior of male moths is stereotypically characterized using the following motions: a straight upwind flight called ``surging'', a narrow zigzagging motion [15], [16], [17], and wide lateral excursions, sometimes called “casting” or “sweeping”. Although it is acknowledged that male moths’ navigation is based on chemical and visual cues [18], the mechanism underlying the source-locating navigation is not fully understood [19]. Nonetheless, different theoretical strategies ([20], see the reviews by Cardé [19,21]) were proposed to explain the navigation mechanisms of male moths. However, the proposed strategies for locating an odor source by male moths vary in their assumptions, orientation mechanism, and in their sensory inputs. Some strategies are based on an endogenous program (termed internal counter, [22], [23], [24]), others are based on external cues guided by the instantaneous changes of the pheromone plumes properties (e.g.: concentration, [25]) or its spatial structure [26], which can additionally be used by memory-based steering (*Manduca sexta*, [27]). Additional steering is based on the local direction of the wind [28,29]. Following these conceptual strategies for odor-based navigation, numerous models were proposed to simulate this phenomenon [27,[30], [31], [32], [33], [34], [35], [36], [37], [38], [39], [40]]). To evaluate the feasibility, accuracy and readiness of the aforementioned moths-navigational models, a unifying framework is necessary. This componential framework will provide a platform used as a benchmark tool. The framework will allow testing the performance of virtual navigators under control conditions of their environment. Recently, Macedo et al. [41] reported on a simulator and comparison of several bio-inspired and engineered strategies for chemical plume tracking. However, this framework was based on the diffusion process without accounting for wind or turbulence that are at the core of the moth-inspired navigation strategies [27,^30^,^36^,37].

Hitherto, we provide a framework using an open-source computational platform considering wind speed and direction and plume characteristics, set as parameters that can be adjusted in order to more realistically simulate the environmental conditions. We examine few available moth-inspired navigation strategies, based on a prescribed wind and plume model.

We examine some of the available moth-inspired navigation models based on two navigational strategies, using a prescribed wind and plume model. The main goal is to provide an accessible and reproducible simulation platform, promoting the development of navigation strategies using olfactory that can be utilized in the design of aerial autonomous vehicles.

## Methods and materials

The comparative framework is based on numerical simulations for odor-based navigation. We utilized the wind and plume models proposed by [42], that implemented in an open-source software package [43]. This model served as a benchmark for the navigation models. These models were simulated using ‘MothPy’, which is an open-source package written in Python and developed by Benneli and Liberzon [44]. In the following section, we briefly review the wind and plume models [42], and in greater details, the four navigation models used herein. The four models can be divided into two conceptually different strategies, while each of the models consists of different parameters. The comparison has been performed at instances where the navigational strategies were similar: casting, zigzagging and surging to enable statistical comparison.

### Computational framework

The computational framework is an open-source package written in Python [44]. It is based on the open source scientific software packages of Numpy, Scipy, Matplotlib and Jupyter. The software package can simulate several classes of the wind, plume [43] and odor-based navigator. For easier adoption and reproducibility, we developed an online cloud-based Jupyter notebook (use the link from the software repository).

### Wind movement and plume dispersal models

**Fig. 1:**
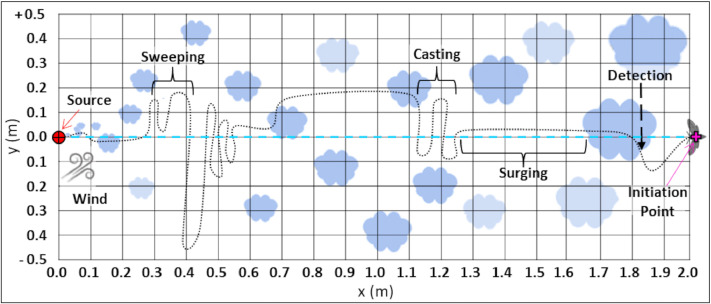
The simulation field. A 2D rectangular grid of 1.0 (y) X 2.0 (x) in metric units. The wind propagated from left to right (increasing values of x). Blue clouds represent the dispersion of the pheromone odor. The odor source is located upwind (left side) at the midpoint of the horizontal plan (x,y: 0.0, 0.0), represented by red circular target. The navigators initiate their flights at the farther downwind area of the field, located at the midpoint of the horizontal plan (“initiation point”: x,y: 0.0, 2.0), represented by the pink plus mark. The blue dashed line represents the central line that connects between both of the horizontal midpoint, i.e. the locations of the source and the navigators' initiation point. An illustrated path represents the hypothetical trajectory of the navigators' flight. The display of the behavioral repertoire of the moth-like navigator is shown (behavioral elements: “detection”, “surging”, “casting” and “sweeping”).

The flow domain of the simulation assumes an imaginary two-dimensional rectangular grid of 1.0 × 2.0 m (see Fig. 1). Each of the virtual flyers in the simulated environement is defined here as a navigator. The mean wind moves from left to right, the plume (contains the odor) source is located downstream at the midpoint of the left side of the grid (horizontal plane: x=0.0 m, y=0.5 m) dispersing with the wind; the navigator starts on the right side of the grid. The simulated flow comprises of two components, the streamwise and the spanwise planes. It simulates a horizontal flow field parallel to the ground at some height above the ground (we assume that the surface is smooth and the presence of obstacles like vegetation, etc.). The two velocity components are the streamwise (i.e. wind primary direction) component, u, and the transverse (i.e. cross-wind) direction, v. Generally, the crosswind component is about an order of magnitude smaller than the streamwise component, but not always negligible. In some cases, when the user may want to simulate turbulent meandering plumes in complex environments, the wind model has the option to include random noise, which represents some similarities to turbulent velocity fluctuations [42]. The simulated wind acts as the forcing function to carry and disperse the odor. In order to allow more realistic conditions, we added a periodic, large-amplitude and relatively slow (in respect to the flight time of a navigator, as will be shown later) component that mimics the meandering of the odor-plume (which is described in the next section) due to gusts. Meandering determines the extent to which the wind changes direction during instances when the odor is released. This setup does not fully replicate real turbulent flows, nor does it account for the complex interactions within the flow due to turbulence. Nevertheless, it creates a similar turbulent-like plume; although this is not a realistic physical model, its strength is in providing a reasonably fast simulation framework for testing multiple navigators’ strategies.

Thus, the mathematical formulation of the wind model prescribed at every location x, y is:

(1) $$\begin{array}{c} \left\{ \begin{array}{c} u\left(t\right)=\;A\;+\;\beta \;\\ v\left(t\right)=\;B\sin\left(\omega t\right)+\;\beta \end{array}\right. \end{array}$$

where A, B, ω are constants, chosen as the simulation parameters for the wind speed, meandering amplitude and period, respectively. The parameter β represents a random white noise. Note that meandering is characterized by two parameters: (i) amplitude (m/sec) and (ii) period (rad/sec), as defined in Eq. (1). In this work, we keep the period constant while varying only the meandering amplitude (parameterized in Eq. (1) as A). For more detailed technical information on the implementation of the wind model, see [42].

### Plume model

The plume model simulates the release of odor (i.e. scalar; [42]) from a point source located upstream with respect to the navigator. Conceptually, the source emits the odor through the so-called “puffs”. The term “puff” defines a concentrated region of a scalar that is advected as a clustered group in the stream-wise direction and experiences collectively, momentum and energy exchanges. The puff releasing rate can be determined according to the user's interest. The boundaries of the puff are assumed to be well-defined. We define the puff as a two-dimensional Gaussian shape carried downstream by the wind. The coordinates of the puff center, *x*_p_(t), *y*_p_(t) and the concentration distribution around the puff, C, which follows a Gaussian function, defines the puff boundaries and intensity. The puff center, *x*_p_(t), *y*_p_(t) moves within the flow field at a speed determined by the wind vector (*u, v*). For simplicity, we use bold mathematical symbols for the 2D vectors, i.e. *u* = 

(2) $$\begin{array}{c} \left\{ \begin{array}{c} \left\{u,\;v\right\},\;\mathrm{xp}\;=\;\left\{\text{xp},\;\text{yp}\right\}:\;\\ \mathrm{xp}\left(t\;+\;\Delta t\right)\;=\;\mathrm{xp}\left(t\right)\;+\;u\left(\mathrm{xp},\;t\right)\Delta t\; \end{array}\right. \end{array}$$

The odor concentration C (r, t) in a single puff is determined by the distance from the puff's center, x_p_(t), as well as by the time passed since the formation of the puff, t. As the puff moves downstream with the wind, it becomes more dispersed. The concentration field can be expressed as follows:

(3) $$\bar{C}\left(x,t\right)=\frac{m_{p}}{2\sigma^{2}}\sum_{i}^{\infty }H\left(t-t_{i}\right)exp-\frac{{\left|x_{p}\left(t-t_{i}\right)\right|}^{2}}{2\sigma^{2}}$$

where m_p_ is the mass of the puff, H is a Heaviside function and σ is the spreading rate proportional to turbulent diffusivity [37]. In the following, we simplify the problem assuming the navigators have a binary sensor, therefore the concentration of odor is translated into the size of the region in which the concentration is above a threshold of detection, i.e. C ≥ C_0_. The size in this approximate model is based on a circular patch of radius r_p_(t)^2^ and its area is proportional to σ^2^:

(4) $${\left(t\right)}^{2}=r_{p}\left(t-t\Delta \right)+\sigma^{2}t$$

Parameters of the odor source are the puff release rate, f_r_ (i.e.: puffs per second) and puff spread rate, dr_p_(t)/dt. The spread rate assumes a linear rate of increase of r_p_(t), as shown in Eq. (4). These two source parameters, together with the wind parameters and the concentration threshold of the navigator's odor sensor, determine the properties of the plume. For instance, setting the threshold to a negligible value will convert the plume type from an array of discrete, concentrated puffs into a single, featureless stream of odor. We are interested in the present case study in a downwind spreading plume of odor, mimicking a trail of puffs, similar to that formed in a wind tunnel where a single female moth is secreting pheromone [45]. For a detailed description of the parameters used in the wind movement model and the plume dispersal model see Table 1.

**Table 1:** Model variables and parameters. The model variables are: A - constant average wind speed, B - constant average meandering amplitude, β - a random white noise, ω - angular rate of change of the wind vector direction, R - transverse diffusion of puffs, f_r_ - puffs release rate, r_0_ - initial radius of a puff formed at the source location, α - rate of puff growth, C_0_ - odor detection threshold of a navigator, $\hat{T}$ - flight time of a navigator to reach the source along the straight path from its initial position.

Parameter	Values	Units
A	1	m/s
B	0-0.15	m/s
ω	0.1	rad/s
β	0	m/s
R	0.5	m^2^/s
f_r_	50-200	1/s
r_0_	0.001	m
σ	0.0002	m^2^/s
C_0_	500-2400	a.u.
$\hat{T}$	1.8-2.4	s

### Navigation strategies

We chose two navigation strategies where each has two different models. These are based on Liberzon et al. [37], named here as: “A”, and on Bau and Cardé [30], named here as: “B”. The core of both strategies is an odor-mediated navigation model of optometric anemotaxis [45,46]. For each strategy, we set specific modifications based on empirical studies, resulting in two navigation models for each of the two strategies: “A_1_”, “A_2_” and “B_1_”, “B_2_”. An overview of each navigational strategy is provided in the following. Note that each type of the two strategies, ``A'' and ``B'', is originally inspired by the behavior of two different moth species, *Cadra cautella* ("A”) and *Lymantria dispar* (“B”).

### Navigation definitions

The strategy of the navigator model is comprised of a set of rules and constraints that underline the decision-making process. For the cases studied here, several assumptions are similar to all strategies:

• The navigator is a free-flying object travelling at a constant ground speed and utilizes a binary sensor (yes/no) for the odor cues.
• The navigator can only measure the local wind direction and it can use an internal counter [24] for the time scale estimates.
• The navigator does not have a long-term memory or spatial information with respect to a fixed coordinate system ('no GPS signal').

The navigator is defined as an object marked by a point in a two-dimensional space, x_p_ ∈ R^2^, a point-sensor of the local wind velocity, u(x), and presence/absence of odor c(x_p_) = 1/0, as shown in Fig. 1. Although a flying navigator will only sense wind velocity relative to itself, we assume that using optometry data, the navigator can find the direction of the wind relative to the ground. Here we adopt the widely accepted notion of optomotor anemotaxis [30,36]. This assumption is in accordance with the directly observed behavior of moths in a wind tunnel and in previously suggested models [37,42]. The (constant) ground speed of a navigator was set to 0.4 m/s^−1^ , in accordance with previous studies performed in a wind tunnel assay [26,47]. The binary sensor threshold of a navigator is the last parameter that defines the field for a given navigator, as shown in Fig. 1. In this figure, regions that would be tagged “detected” puffs are marked by white pixels, and the background (the concentration below the threshold) is dark. It is noteworthy to mention that physically identical plumes (same wind, turbulence, release rate, concentration) may not appear the same to different navigators depending on their detection threshold. We address this issue in the results and the discussion.

A navigator is initially placed at the releasing position (x_0_, y_0_) downstream relative to the source (x_0_> 0) within an area that has a certain probability to encounter a puff. For all navigators, the odor-mediated flight is based on optomotor anemotaxis mechanism. Hence, the navigator is using visual information to evaluate the local flow direction in order to fly in the upwind direction. Note that the steering mechanism does not involve any neural processing in this simulation.

The navigation starts when a puff with a concentration above a given threshold “reaches” the initial location of the navigator. This moment is marked as the initial time of the navigation path t_0_. The behavior of odor-mediated navigation includes a repertoire of elements:

The navigation path consists of several possible time intervals:

• “detection” - the time of flight during which the navigator is inside a puff, i.e. the measured concentration is above the threshold;

• “surging” - straight upwind flight after the detection interval;

• “casting” or “zigzagging” - crosswind flight with alternating changes of direction, typically when the signal has been recently lost;

• “sweeping” - large random motions that are designed to increase the probability to encounter an odorant signal.

**Fig. 2:**
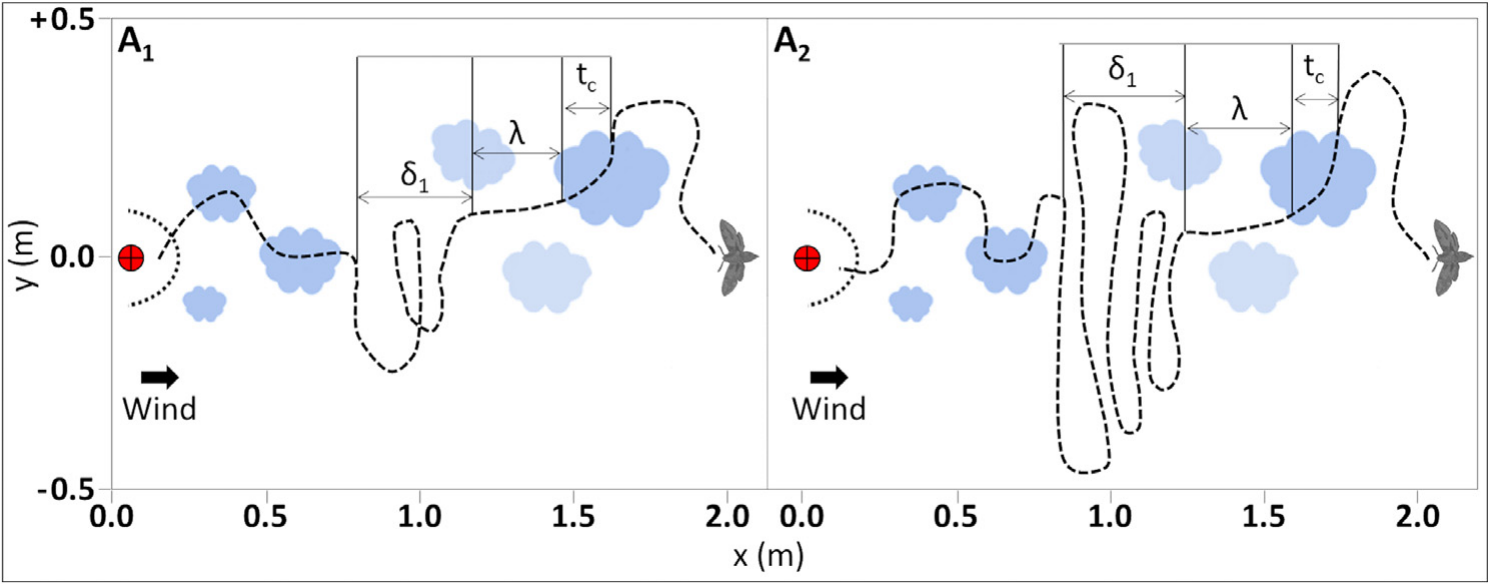
Schematic description of both types of navigation models of strategy “A” [37]. Left - model A_1_, Right - A_2._ The mean wind direction is from left to right. Due to the turbulent nature of the odor spreading and diffusion, pheromone puffs are increasing in size with the distance from the source. Red rounded target - the position of the source. Dashed crescent - the area of success, radius of 0.15 m from the source's center. Blue plumes - the pheromone puffs. In both models the navigation prototypes are based on the “detection time": at the time of detection of the odor, the time of crossing is defined as t_c_ and used for the following surging (upwind flight for time denoted as λ and casting (crosswind flight, a typical time denoted as δ_1_). For model A_1_, casting (δ_1_) is mathematically defined as α(t_c_), where α is a predefined coefficient equal to 1.5. In model A_2_, casting (δ_1_) is mathematically defined as (α(t))(t_c_), thus this type of navigating behavior is characterized by relatively prolonged casting.

**Fig. 3:**
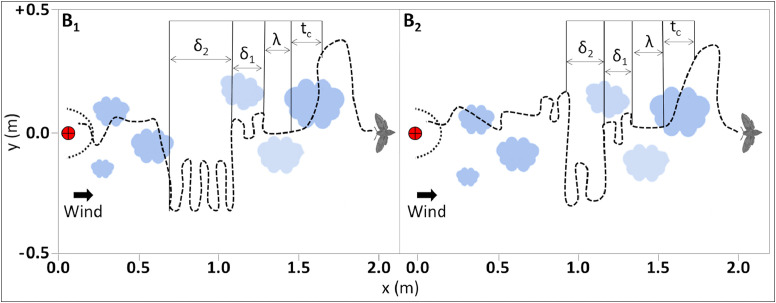
Schematic prototype of both types of navigation models of strategy “B" following [30]. Left - model B_1_, Right - B_2._ The descriptions of the “mean wind” direction, the spreading of the pheromone puffs and the position of the source are similar to those mentioned above in Fig. 2. In both models, the navigation prototypes are based on predetermined constants given for the behavioral elements of surging (λ) and casting (δ_1_). Contrary to strategy “A” (see Fig. 2), both models of strategy “B” have an additional behavioral element named “sweep” (δ_2_), which follows the display of casting behavior. The sweeping behavior differs between the two models in the aearance rate and the amplitude of this element. For example, the navigation model characterized by a large motion of cross-wind direction at time scale δ_2_, alternating with the casting behavior (small zigzags) at the time scale δ_1_.

We summarize the key parameters of the navigators in Table 2. Both strategies are based on an essential parameter: the puff crossing time, defined here as “detection time”. This will be denoted as t_c_ and it resets every time a navigator crosses a puff. We will use the same notation for all the navigation strategies. After detection, an elapsing time, λ, will be spent for surging (fast upwind motion according to the local wind direction), see Fig. 2. In strategy ``B'', the time, λ will be a constant (predefined time interval), see Fig. 3.

**Table 2:** Key parameters of the navigators simulated in this study.

Strategy	Model	Surging	Casting	Parameter	Sweeping
		λ	δ1	α	δ_2_
A	1	t_c_	αt_c_	1.5	None
A	2	t_c_	α(t_c_)	α(t)	None
B	1	λ	δ1	1	7δ_1_
B	2	λ	δ1	1	3δ_1_

These elements of the navigation strategy are similar to those observed in moth flights, see e.g. [30]. For both models of strategy ``B'', λ, δ_1_, are predetermined constants.

### Strategy A

Strategy “A” is a temporal-based model that relies on sequential comparison of the elapsing time it takes the navigator to cross the plume (see below ``detection time'', t_c_). Here, if the navigator does not meet a new puff within the time of surging λ, it will start casting using transverse zigzags during time intervals, denoted as δ_1_. For strategy ``A'', the definitions of both of the behavioral elements: surging (λ) and casting (δ_1_), include the parameter α which can be varied. Strategy ``A'' is different from strategy ``B'' in two main aspects (see below the description of `Strategy B'): i) both surging and casting are not predefined as constants; and ii) strategy ``A'' is simpler than strategy ``B'' because it does not consider sweeping (δ_2_).

### Model A_1_

Navigator “A_1_” (Fig. 2) determines its path based on the previous detection time, λ = t_c_. For this navigation model, the time interval of casting (δ_1_) is proportional to the time interval of surging (λ), i.e.: equal to the last detection or crossing time t_c_, hence λ = αt_c_. In the studied case shown here, α = 1.5.

### Model A_2_

Navigator “A_2_” features a small modification as compared to “A_1_”. In strategy “A_2_”, casting time increases with every other turn, slowly growing and covering a larger cross-wind width. It has δ_1_ = (α(t))(t_c_), where α(t) marks a continuously growing function with a predefined coefficient. A typical flight path of the navigator type “A**_2_**” is shown schematically in the left subplot of Fig. 2.

### Strategy B

The flying program of strategy B is based on predetermined constants, λ, δ_1_: after every detection, the navigator surges upwind for a predetermined time (λ). When the navigator cannot detect any odor trail after a predefined time interval λ, it changes to casting mode using another predefined time interval δ_1_. After several iterations (an arbitrary number of turns that is defined following the literature, see below in `Model B_1_' and `Model B_2_'), the navigator will perform a large sweep, as shown in Fig. 3. The time interval, which characterizes the sweeps, δ_2_, varies between the two models: “B_1_” and ``B_2_”. This parameter can differentiate the success rate of flyers with large sweeps versus small ones. Note that there is an additional parameter in the sweeping behavior, where the sweeping angle is randomly chosen with respect to the direction of the wind. Thus, a sweeping behavior has a probability of 50:50 % in either redetecting or distancing the plume. In contrary to strategy ``A'', both λ and δ_1_ are constant (predefined time interval).

### Model B1

Following the literature (Willis and Baker, 1988), the time interval for this model is set to 7, i.e. δ_2=_ 7δ_1_.

### Model B2

Following the literature (Kuenen and Cardé, 1994; Willis et al. 1991), the time interval for this model is set to 3, i.e. δ_2=_ 3δ_1_.

### Simulation performance

Each group of navigators is placed at the same starting points of the simulation field before release. In each model, the effect of the two indirect variables (e.g. increased meandering amplitude and puffs' releasing rate) on the flight performance of the navigators is tested. Each indirect variable comprises four different treatments of increasing values: meandering amplitude (A: 0.05, 0.1, 0.15, 0.2 [m/sec]) and puffs releasing rate (σ: 0.0001, 0.0002, 0.0003, 0.0004 [m^2^/sec]).

For each treatment (8 in total), multiple navigators (*n*=250) of each of the four models (1000 navigators in total) are released in sequential iterations. In each iteration, the value of the independent variable is re-modified to one of the four chosen values (thus, each treatment comprises 4 iterations). To prevent any dependency, the navigators are independent and cannot interact with each other. In total, each model includes 8000 navigators.

### Statistic and analysis

The investigation includes different levels of comparisons for different goals: First, under the arbitrary values chosen, it is possible to investigate the performance of the two navigation strategies that are distinguishably different concerning the spatial-temporal perspective. While strategy ``A'' is based on temporal-based (i.e. ``detection time''), strategy ``B'' is spatially-based. Second, a pairwise comparison between two models of the same strategy allows characterizing the effect of specific modification on the flight behavior of the navigator. Third, a comparison among all four navigation models allows testing the effect of specific modifications to the flight strategy. For example, the effect of the increasing width of the casting (strategy A: A_1_ vs. A_2_), or the effect of the rate of sweeps (strategy “B”: B_1_ vs. B_2_). To test the navigators’ ability to find the plume source, we used two independent variables, following the bio-statistical approach commonly used in behavioral ecology of flying insects [48], [49], [50], [51], [52] and plume-tracking algorithms [31,53]:

(1) Success rate - is the percentage of navigators that reached the origin (within a short distance of 0.15 m). Navigators which did not encounter any odor, and consequently did not begin their search, were omitted from this calculation.

(2) Efficient navigation time, τ (ratio) - the average ratio between the time of a navigation and the minimal theoretical navigation time, as expressed by: τ = T/ $\hat{T}$. $\hat{T}\;$is defined as the total navigation time of a successful navigator that elapsed from the beginning of the flight until reaching the odor source location. T is the shortest distance flight path of the successful navigation flight, divided by the ground speed (i.e.: 0.4 m/s^−1^). Only successful navigation paths are considered. It is a measure of the navigation efficiency: a smaller ratio can be interpreted as a more efficient navigation algorithm.

We test the effect of two independent variables; (i) the meandering amplitude; and (ii) the puff spread rate on both the (1) success rate and the (2) efficient navigation time, for all four navigation models. We followed the bio-statistical approach commonly used in ethological studies [54]. In order to perform a comprehensive investigation, (known as full factorial analysis) we performed a statistical analysis which includes two factors: (1) the effect of the independent variables (i.e. physical variables); and (2) the effect of the navigation model. This analysis is comprised of two parts: the navigator ability to allocate the odor source and the time it takes the navigator to find the source.

In the first part, we investigated the success rate of the navigators in locating the odor source; as this index is comprised of discrete data (i.e. counts of binary data), we analyzed it using the statistical family of contingency tests [55,56]. The comparison of the success rate among all four navigation models was done in a three-step methodology. In the first step, we investigated the difference among treatments using the two-sided Fisher exact test for multiple comparisons (df=15). This test is more suitable than the alternative Chi-Square test of independence, when more than 20% of the expected frequencies of the table-cells are below the value of five [57]. In the second step, this procedure is followed by pairwise comparisons using the two-sided Fisher exact test (df=1) with the Holm-Bonferroni method. This post-hoc analysis allows to test the difference between each pairwise. In the third step, we tested the within-group effect (all four models in the same sub-group of the independent variable) using the G^2^ test of goodness of fit [58], following the null hypothesis of uniform distributions of all navigation models in each of the sub-treatments. In the second part, we tested the effect of each of the two independent variables (the meandering amplitude or the puff spread rate) on the efficient navigation time using a two-step methodology. First, we used the non-parametric two-way analysis of variance Scheirer-Ray-Hare test (SRH). This test is the alternative method to the parametric test of the two-way ANOVA. Yet, because our data do not match the assumption of normality, (Shapiro-Wilk test, *p* < 0.05 in all four navigation models) we used the non-parametric alternative. We investigated the difference among treatments using the two-way analysis of variance Scheirer-Ray-Hare test. Then, between-analysis was done using the post-hoc comparisons of Dunn's test. This statistical method is commonly used as a non-parametric alternative to the full factorial two-way ANOVA. All of the statistical analysis was done by using JMP®Pro 14 [59] and the R programming language, version 3.5.1 [60].

## Results

### Simulated plume dispersal

**Fig. 4:**
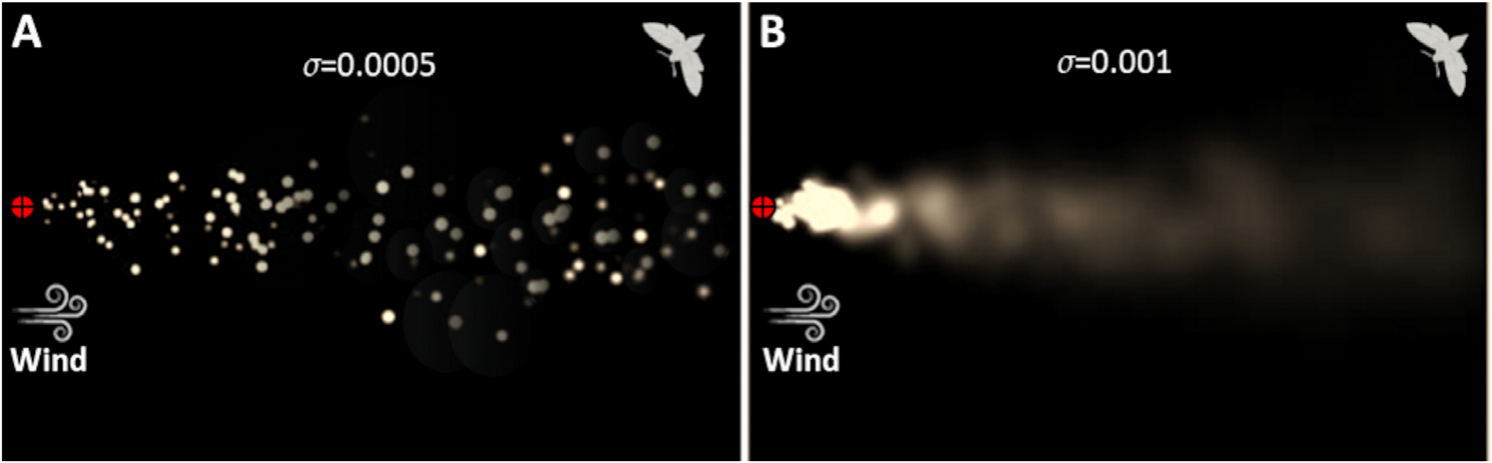
Simulated plumes. Two examples of the simulated plumes with discrete puffs, based on the model of [36], using [43]. The wind direction is from left to right and the source (“female moth”) is at the origin located at the centre of the left side of the figure. The simulated plume is for two different spread rates (σ, m^2^/s): (A) a plume of separated puffs (σ= 0.0005), and (B) a continuous plume (σ= 0.001). For A, the colors of the puffs demonstrates the threshold limit of the navigator binary sensor: bright puffs represent concentration above a low threshold (1500, arbitrary units) and gray puffs represent concentration above a high threshold (30000, arbitrary units).

The simulator can mimic different plume types: laminar or turbulent, continuous or sparse, patchy , and strong winds that can have strong gusts or meandering. Visual outputs of the embedded odor-dispersal model are shown in Fig. 4. The plots provide an example for the specific modification that can be done by the user. For instance, we adjusted the physical environment by modifying the puffs releasing rate (denoted by σ) to a low releasing rate (σ=0.0005, Fig. 4A) or a high releasing rate (σ=0.001, Fig. 4B).

### Simulated flying navigators

**Fig. 5:**
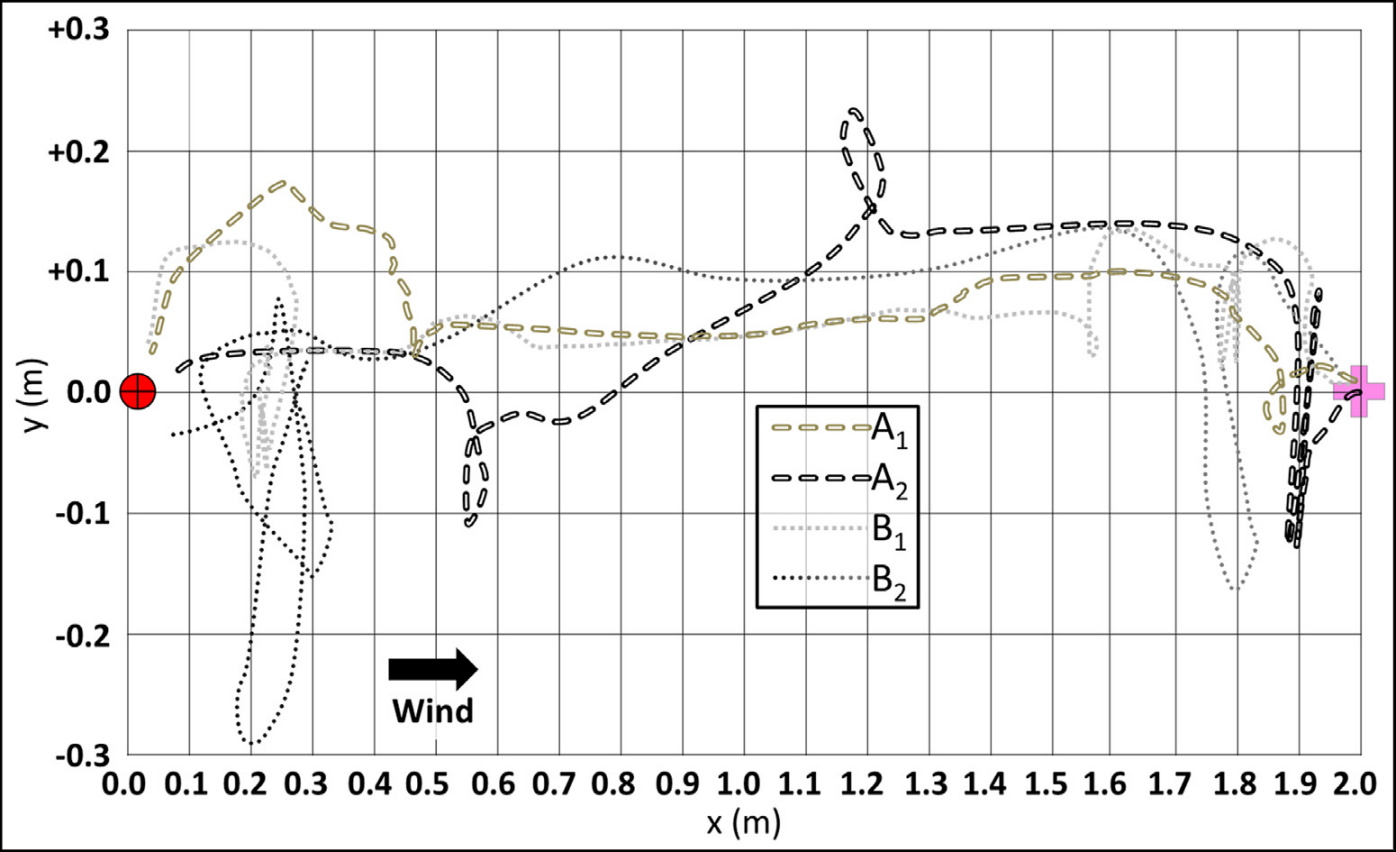
Stereotypical flight path of each of the four navigation models. The rectangular grid represents the coordinate system of the simulation field in metric units. The general wind direction is from left to right. The navigation started at large x_0_ (x_initiation point_= 2.0, right side) towards the origin of the source, x_source_ = 0 (left side). All the navigators have the same ground speed.

**Fig. 6:**
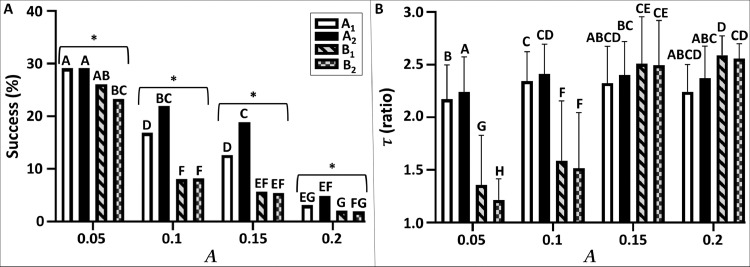
Flight performance of different navigation models under varying levels of meandering amplitude. The two model types of strategy ``A'' (A_1&2_) are shown by the clear bars, where those of strategy ``B'' (B_1&2_) are shown in a geometric pattern. A, the success rate (%) of navigators in reaching the vicinity of the source. A comparison among all treatments across all four navigation models was done using the two-sided Fisher exact test for multiple comparisons; pairwise comparisons were done using the two-sided Fisher exact test following the Holm-Bonferroni adjustment method for α = 5%, where a significant difference between two bars is indicated by different capital letters. A specific comparison among all four models (denoted by '---'), in each of the four sub-treatments (i.e. the level of meandering amplitude, A: 0.05-0.2) was done using the G^2^ test of goodness of fit; * indicates a significant difference at *p* < 0.05. B, the efficient navigation time (τ). Comparison among all treatments across all four navigation models was done using the Scheirer-Ray-Hare test following post-hoc comparisons of Dunn's test; a significant difference between a pair of models is indicated by different capital letters. Common letters indicate for insignificant difference.

**Fig. 7:**
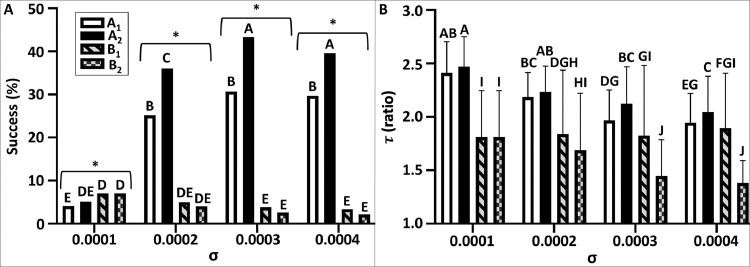
Flight performance of different navigation models under varying levels of puff spread rate (σ) under turbulent conditions. Navigation models and statistical comparisons are similar to the description above in Fig. 6. A, the success rate (%). B, the efficient navigation time (τ).

The results in Fig. 4 also show how the interplay between a navigator and its physical environment is controlled by the user. The simulation plots visually show two cases of navigators that are characterized by a different detection threshold, either low (Fig. 4A) or high (Fig. 4B). The performance of virtual moth-like navigators is shown in Fig. 5, presenting the flight paths (randomly chosen) of each of the four navigation models. A successful navigation ends in the proximity of the origin x = 0, y = 0. In the following, we present results of the simulated investigation, where the effect of two abiotic parameters on the moth-like navigators was tested: (1) meandering (Fig. 6), and (2) the puff spread rate (Fig. 7).

### Effect of physical parameters on the navigators

We tested the influence of two physical independent variables; (i) the level of meandering amplitude (A, Fig. 6); and (ii) the releasing rate of the odor puffs (σ, Fig. 7) under turbulent conditions.

Both physical parameters had a significant effect on the flight performance of the navigators (two sided Fisher exact test for multiple comparisons, df=15, *p*< 0.001 for both, Figs. 6A7A). It appears that the task of successfully reaching within the vicinity of the source ('success rate') by the navigator was more susceptible to the effect of the meandering level (Fig. 6A) than the effect of the releasing rate (Fig. 7A). The effect of the meandering amplitude (Fig. 6A) depicted a clear tendency by which increasing values of meandering reduced the ability to locate the source in all models. In both of the extreme conditions of meandering amplitude (minimum or maximum: *A* = 0.05 or 0.2, correspondingly), all navigation models were very successful at the lowest meandering values (*A* = 0.05: success rate: min-max, 23.3–29.1%), or very unsuccessful at the highest meandering amplitude (*A* = 0.2: success rate: min-max, 1.9–4.8%). Nevertheless, both navigation models of strategy “A” were more successful than the models of strategy “B”, regardless of the level of the meandering amplitude. The most prominent differences among the four models was found in the intermediate values of the meandering amplitude (*A*: 0.1, *G*^2^=159.31, df=3, *p* < 0.0001; 0.15, *G*^2^=116.17, *df*=3, p <0.0001). Contrarily, a lower inter-models difference was found in the extreme values (*A*: 0.05, *G*^2^=8.64, *p* < 0.05; 0.2, *G*^2^=17.47, *p* < 0.001). Contrary to the negative effect of the increasing level of the meandering amplitude, a higher releasing rate of puffs (σ) had a positive effect on the ability of the navigators to reach the source (Fig. 7A). Strategy “A” appears to be more successful (specifically model *A*_2_) through the simulation for this set of parameters. Furthermore, the releasing rate had a varying effect on the success rate of all navigation models, among all sub-treatments (σ= 0.0001–0.0004). Notably, while the inter-model difference is lower in the lowest releasing rate (σ= 0.0001, G^2^=11.09, p < 0.05), it is profoundly higher in all other sub-treatments (σ: 0.0002, *G*^2^=382.54, *p* <0.0003; *G*^2^=704.38, *p* < 0.00; 0.0004, *G*^2^=745.98, *p* < 0.001). Particularly, the success rates of both models of strategy “B” were relatively low, in all levels of the releasing rate (min-max: 2–7%). It is worth mentioning that at the lowest releasing rate (σ= 0.0001), both models of strategy “B” were higher than their counterparts (B_1_, B_2_ vs. A_1_, A_2_: 7.0% for both vs, 4.1%, 5.1%, correspondingly). In addition to the success rate, we compared the level of efficiency (τ) of all the successful navigators under the influence of the meandering amplitude (A, Fig. 6B) and the puffs releasing rate (σ, Fig. 7B). The 2-way factorial comparisons (i.e. Scheirer-Ray-Hare test) showed a significant effect of both indirect variables (A & σ) on the flight efficiency of the virtual navigators; between the two indirect variables, the impact of the meandering amplitude was more eminent than the puffs releasing rate (*A, H*=384.00, df=3, *p* < 0.0001; σ, *H*=83.65 df=3, *p* < 0.0001). The full factorial model revealed that the navigation model had a significant impact on the flyers, in both types of physical conditions. However, the role of the navigation model was more decisive when coping with different types of puffs' releasing rate, but lesser when facing different types of meandering amplitudes (*A, H*=516.44, df=3, *p* < 0.0001; σ, *H*=310.07 df=3, *p* < 0.0001). In both type of strategies, a significant interaction was found between the indirect variables (*A* & σ) and the navigation model ("A_1_","A_2_" & “B_1_", “B_2_”) (*A, H*=250.26, df=9, *p* < 0.0001; σ, *H*=33.77 310.07 df=9, *p* < 0.001). Both models of strategy “B” were significantly more efficient than their counterparts at the lowest level of the meandering amplitude (*A*=0.05 & 0.1, Dunn's post-hoc test, *p* < 0.05). Contrarily, both navigation models of strategy “A” were more efficient at all levels of the puffs releasing rate.

## Discussion

Biologically-inspired algorithms have attracted increasing interest as a biomimicry tool in the scientific research of chemical sensing [30,61]. From the bio-engineering perspective, bio-inspiration algorithms of localization of odor sources can be used for different proposes, either as complementary in the experimental study, or in applied science [61], [62], [63].

A common computational framework will provide a comprehensive numerical tool that will enable to compare the performance of moth-inspired navigation algorithms. In this work, we developed a computational framework of a self-propelled navigator, inspired by the odor-mediated navigation of flying male moths. It is an open source package (Python, `MothPy', see [44] providing a benchmark framework of simulated navigation strategies. Herein, we implemented four navigation models based on two conceptual strategies, inspired by two moth species [30,37], and the open source package (“pompy”) of the puff-based odor plume model [42].

This platform enables a quantitative comparison between various odor-based navigation concepts. In this study, we compared between two conceptual bio-inspired navigation strategies: a simple navigation behavior that based on temporal sampling of the odor plume (“strategy A”), and a navigation behavior that is more conserved (based on predefined constants), with a richer repertoire (“strategy B”). Our results showed that the flight performance of the virtual navigators, under different physical conditions, is strategy dependent. For instance, strategy “A” is favored in successfully locating an odor source, in both types of turbulent conditions (Figs. 6A, 7A). However, strategy “B” is a more efficient searcher. This may infer that a hybrid model of both types of strategies can generate an optimum strategy for an autonomous navigator. Another important aspect in the quality assessment of self-propelled navigators is listing the limitations of the investigated strategy. Based on this case study, the effect of the meandering level appears to be more significant than the effect of the puff's releasing rate. Finally, although our simulations were not intended to simulate a real male moth, they provided insights that are at the core of behavioral ecology of male moths ("risk-averse vs. risk-tolerant behaviors", [27]. For instance, as a male moth searches for a female, he has a tradeoff between being the first to reach her (success) and his searching efficiency (less energy expenditure). This success-efficiency tradeoff is manifested in the flight performances of the two conceptual strategies tested, “A” (high success & low efficiency) and “B” (low success & high efficiency). These insights may extend the applicability of our benchmark framework also as a complementary tool in the study of sexual communication in moths. Simulated studies are heuristically used to investigate a different facet of the odor-mediated mate finding in moths. For instance, [30] conducted a simulated study to compare the performance of moth-inspired navigators, (based on the flight characteristics of the gypsy moth, Lymantria dispar) among different theoretical strategies of the animal's movements. Also, a recent work [64] has shown the feasibility of using our proposed simulation framework, in aspects regarding sexual communication in moths. Stepien et al. [64] generated agent-based numerical simulations to investigate specific aspects of the sexual reciprocity in moths, with a special emphasis on the females' calling strategy and the males' navigation strategy. Simulated modeling may contribute in shedding light on the evolutionary dynamic of moths, and their behavioral ecology.

The growing research area of computational bio-inspired plume-tracking has tremendous applicable potential. The development of highly efficient plume-seeking robots can be used for different tasks related to precision agriculture, security, military and more [65], [66], [67], [68], [69]. Besides, a computational benchmark can be used as a complementary tool for the empirical study of plume-following organisms, a field that is known for its high complexity (biological, physical and chemical).
